# Principles for Rigorous Design and Application of Synthetic Microbial Communities

**DOI:** 10.1002/advs.202514750

**Published:** 2025-12-20

**Authors:** Yuxiao Zhang, Minyu Jing, Lihui Lyu, Li Nie, Xihui Xu, Ru Sun, Xiyuan Xu, Siyu Chen, Shuobing He, Yumeng Zhang, Ping Huang, Weijie Luo, Jiaojiao Liang, Guifeng Gao, Kunkun Fan, Teng Yang, Liyan Zhang, Xiao Fu, Sarah M. Allard, Jack A. Gilbert, Jiabao Zhang, Haiyan Chu

**Affiliations:** ^1^ State Key Laboratory of Soil and Sustainable Agriculture Institute of Soil Science Chinese Academy of Sciences Nanjing China; ^2^ College of Life Sciences Nanjing Agricultural University Nanjing China; ^3^ University of Chinese Academy of Sciences Beijing China; ^4^ Department of Pediatrics School of Medicine University of California San Diego La Jolla USA; ^5^ The Soil Health Center Scripps Institution of Oceanography University of California San Diego La Jolla USA

**Keywords:** application scenarios, design and construction, ecological functionality, interaction mechanism, SynComs

## Abstract

Synthetic microbial communities (SynComs) are microbial consortia with defined taxonomic and functional traits, so that the combination elicits a predictable response under defined conditions. SynComs are artificially designed to enable inter‐species metabolic interactions, metabolic division of labor, and ecological interactions that can elicit phenotypes like colonization stability and environmental adaptation. As an applied tool, SynComs have been deployed in diverse contexts, including agriculture, industry, and environmental ecology. This systematic review explores the processes used to construct SynComs, the mechanisms of metabolic interaction between members, and a review of the different ways that SynComs have been applied. We also explore the challenges for SynCom development and application, and future research directions that could overcome these challenges. SynComs are a powerful tool in our arsenal of applied technologies, but research and application are still nascent. While advances have been made, more research is needed to ensure SynCom technologies do not threaten global ecological security. SynCom technology represents a versatile platform for the controlled manipulation of microbial systems, enabling targeted modification of ecological and physiological processes. This emerging field marks a transition from descriptive biology toward a predictive and engineering‐driven framework for understanding and shaping living systems.

## Graphical abstract

SynComs are artificially designed to enable inter‐species metabolic interactions, metabolic division of labor, and ecological interactions that can elicit phenotypes like colonization stability and environmental adaptation. This systematic review explores the processes used to construct SynComs, the assessment of the mechanisms of metabolic interaction between members, and a review of the different ways that SynComs have been applied. We also explore the challenges for SynCom development and application, and future research directions that could overcome these challenges.

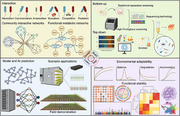

## 1 Concepts, Design and Construction

### 1.1 Key Concepts

**FIGURE 1:**
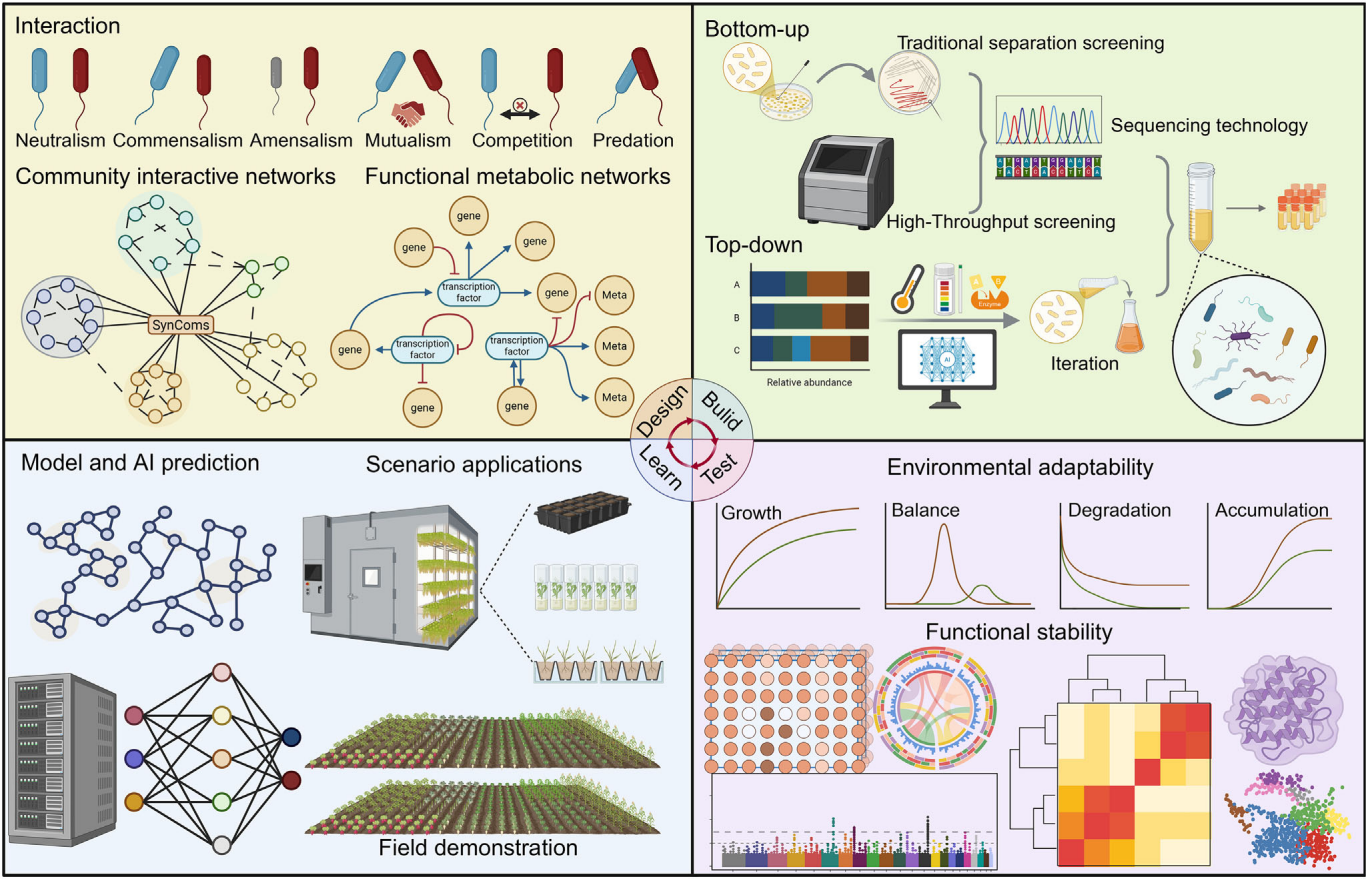
The workflow diagram for design principles‐construction strategies‐test validations‐learn refinements (DBTL cycle) of SynComs.

Synthetic microbial communities (SynComs) (Table 1), also referred to as engineered or designed microbial consortia, are deliberately constructed assemblages of multiple microbial strains, which are usually taxonomically and functionally defined [1, 2]. SynComs are generally designed to fulfill a specific phenotypic role, either to transform particular compounds or promote the growth of another species. The multiple microbial taxa within a SynCom are often chosen to facilitate a functional division of labor, whereby one organism performs a task that can be enabled or supported by other members of the SynCom. This interspecific communication enables a SynCom to perform complex tasks beyond the capabilities of individual taxa. SynComs provide a controlled framework for dissecting the principles governing microbial community assembly, stability, and emergent properties, thereby revealing host–microbe and microbe–microbe interaction mechanisms that can inform the rational design of communities for applications as diverse as soil health, plant growth, bioremediation, and human health. SynCom design is integrated with computational and theoretical modeling frameworks, centered on the design‐build‐test‐learn (DBTL) cycle, which can be used to iteratively improve the desired outcome of a SynCom for a specific situation, as well as enable deeper investigation of the mechanisms of action underpinning a specific outcome (Figure 1) [3, 4].

**TABLE 1:** The terms and definitions provided in this study.

Category	Term	Definition
Basic concepts	Synthetic Microbial Community	An artificial ecosystem comprising two or more precisely identified microbial strains assembled under controlled conditions.
	Wild‐type strains	Microbial strains isolated from natural environments without intentional genetic modification.
	Division of labor	A cooperative strategy where distinct microbial strains within a community execute specialized, complementary subtasks to collectively achieve complex functions.
	Genetically modified microorganisms	Microorganisms whose genetic material has been deliberately altered through genetic engineering techniques.
	Colonization stability	The capacity of a microbial community to maintain its taxonomic composition and abundance profile over time.
	Environmental adaptation	The ability of microorganisms to modify their physiological, metabolic, or structural characteristics to survive and proliferate under changing environmental conditions.
	Microbial community assembly	The ecological process determining which species from a regional pool establish in a specific local community and how they organize.
	Multi‐kingdom consortia	Microbial communities incorporating members from distinct biological kingdoms (e.g., Bacteria, Fungi, Archaea).
Construction	Top‐down	An approach for constructing synthetic microbial communities through systematic simplification of complex natural assemblages.
	Bottom‐up	A construction methodology involving rational combination of axenic cultures to assemble synthetic communities de novo.
	High‐throughput sequencing	Technologies enabling rapid parallel sequencing of numerous DNA fragments for analyzing community composition and genetic potential.
	Multi‐omics analyses	Integrated examination of microbial communities through combined datasets from genomics, transcriptomics, metabolomics, and other omics fields.
	Machine learning	Computational techniques employing algorithms to identify patterns in data and generate predictions, applied to understand and engineer microbial communities.
	High‐throughput pure culture technology	Automated methodologies enabling simultaneous cultivation and isolation of numerous microbial strains.
Ecological interactions	Predictable response	The capability to accurately anticipate microbial community reactions to environmental changes or perturbations based on established rules or models.
	Cross‐feeding	A cooperative interaction where metabolic byproducts from one microorganism serve as nutritional substrates for another.
	Competition	Microbial interactions arising from simultaneous demand for limited resources (e.g., nutrients, space).
	Antagonism	An interaction where one microorganism inhibits or eliminates others through production of antimicrobial compounds (e.g., antibiotics, bacteriocins).
	Cascade degradation	The coordinated breakdown of complex compounds through sequential metabolic steps performed by multiple microorganisms.
	Symbiotic facilitation	A process where one organism's activities create improved environmental conditions for another organism's survival.
Others	Auxotrophic bacteria	Mutant microorganisms incapable of synthesizing particular growth factors (e.g., amino acids, vitamins) and thus requiring external supplementation.
	Niche	The multidimensional functional role and environmental position of a species within a community, encompassing resource utilization and habitat space.
	Microbial community diversity	The variety within microbial communities quantified through species richness (number) and evenness (distribution of abundances).

### 1.2 Design Principles

Currently, the design and construction of a SynCom is informed by four key concepts: (i) functional parsimony and modularization, (ii) prediction and control of interaction networks, (iii) predicting and facilitating a robust and stable community, and (iv) reducing complexity through simplification [1, 2, 3]. First, complex ecological functions are deconstructed into discrete modules, such as resource or environmental sensing, and metabolic inputs and outputs, with dedicated functions assigned to specific microbial strains. This requires a well‐characterized set of taxa so that we can effectively predict these functional modules and reduce unnecessary replication of function. Second, by predicting how functional modules interact, we can effectively tweak and control metabolic processes, signal transmission (such as quorum sensing (QS)), and growth, so as to influence ecological states in the SynCom, such as competitive inhibition between members and further ensure the parsimonious division of metabolic labor. Hence, by predicting and validating interaction networks, we can design SynComs to have precise outcomes in defined conditions. Third, we can engineer communities to ensure their stability across defined environmental stressors; this can be achieved by engineering dynamic feedback control between members and including necessary metabolic redundancy to facilitate long‐term functional and compositional stability. Finally, SynCom engineering usually aims to generate a minimal viable consortium with validated core functionality. This embodies the principle of parsimony, whereby auxiliary strains are incorporated only as needed to address specific challenges related to stability, efficiency, or resistance. Embracing these four concepts in SynCom engineering is what separates SynComs from a straight enrichment of a natural community to achieve a specific phenotype. While we can, of course, use an initial enrichment step to help identify organisms from a wild community for inclusion in a SynCom, we would not refer to the enriched community itself, even if it has a desired activity, as a SynCom.

### 1.3 Construction Methodology

The engineering and construction of SynComs can be broadly categorized as either “top‐down” or “bottom‐up” (Table 2) [4]. The ‘top‐down’ approach is similar to that inferred in Section 1.2 as it usually starts with an enrichment of a community from a wild ecosystem. Natural microbial communities are subjected to environmental conditioning to enrich for functional consortia exhibiting specific functional phenotypes [5]. This is an important step if you do not have an existing set of well‐defined, isolated microbes with which to start with, as you can use natural (or synthetic) selection to identify novel taxa with uncharacterized mechanisms and metabolisms [6]. The “top‐down” approach generally uses strategies such as artificial selection, directed evolution, and then assembly of specific strains [7]. One example involved gradually increasing the concentration of various polycyclic aromatic hydrocarbons (PAHs) in petrochemical wastewater, following which the identified members were isolated and combined to construct a PAH degrading SynCom [8]. In another example, using CO stress in vitro enabled the isolation of a CO‐tolerant chain‐elongating strain of *Pseudoramibacter*, as well as a homotrophic acetate‐producing bacterium *Clostridium luticellarii*, that could be artificially combined post‐isolation to create a SynCom that converts lactate or acetate into C6 carboxylates [9, 10]. Finally, a SynCom capable of promoting plant growth during salt stress was obtained through domestication of a microbial community under salt stress in vitro, followed by assembly [11]. This “top‐down” approach allows the identification and isolation of microbial partners that respond to the same pressure, thereby preserving natural interaction networks. These natural networks arise through the evolution of ecological dependencies, which can help a SynCom to form a stable community. However, due to current limitations in predicting functional and taxonomic characteristics from genes or protein sequences, it remains challenging to elucidate the functional mechanisms that underpin community stability or phenotypic activities.

**TABLE 2:** A comparison of “top‐down” and “bottom‐up” approaches for SynComs construction.

Feature	"Top‐down”	"Bottom‐up”
Core principle	Starts with a complex natural community and applies selective pressure to enrich for a desired function.	Starts from isolated microbial strains and rationally assembles them into a defined consortium.
Construction foundation	Microbial community interactions—complex environmental inocula (e.g., soil, water, rhizosphere).	Individual microbial functions—a collection of well‐characterized, pure microbial isolates.
Key methodologies	Environmental conditioning, artificial selection, serial passage, directed evolution.	Genome‐scale metabolic modeling, computational inference of interactions, rational design based on metabolic complementarity.
Preservation of Natural Interactions	**High**. Leverages co‐evolved, native interaction networks.	**Low**. Includes only the interactions that are designed or predicted, potentially missing complex, emergent properties.
Community Stability	**Typically High**. As it is forged by environmental selection and pre‐existing ecological dependencies.	**Variable**. Stability is a design goal but can be compromised by unforeseen competitive or inhibitory interactions.
Mechanistic Insight	**Low**. Often a “black box”; the functional mechanisms underpinning community performance may be unknown.	**High**. Aims for a “clear box” system where function is based on the known traits of the constituent members.
Control & Predictability	**Low**. Limited control over the final taxonomic composition; outcome is often unpredictable and difficult to reproduce.	**High**. Offers precise control over membership and the potential to predict metabolic outputs and interactions.
Primary Advantages	• Discovers novel, uncultivated taxa and uncharacterized mechanisms. • Yields ecologically robust communities. • Lower technical barrier to initiation.	• Enables rational design for precise functions. • Highly reproducible and defined. • Ideal for hypothesis testing and mechanistic studies.
Primary Limitations	• Causal mechanisms are obscure. • Poor reproducibility and standardization. • Difficult to engineer specific traits.	• Limited by the diversity and characterization of available isolate collections. • May create unstable communities due to simplified interactions. • Resource‐intensive in design and validation.
Typical Applications	• Bioremediation of complex pollutants. • Agricultural inoculants for stress resilience. • When target microbes are uncultivable.	• Production of high‐value biochemicals. • Investigating fundamental ecological principles. • Developing defined probiotics/therapeutics.

Propelled by advances in high‐throughput sequencing, multi‐omics analyses, and machine learning, the concept of constructing SynComs from the ‘bottom up’ is becoming increasingly technologically feasible [12]. By using computational models or predicting network interactions for existing well‐characterized taxa, it is possible to propose a SynCom in silico with defined metabolic inputs and outputs, that can then be validated in vitro and in vivo if appropriate [13]. This facilitates the rational design of SynComs based on metabolic complementarity, making it ideal for defined systems with well‐understood functional mechanisms. For example, a SynCom including *Clostridium cellulolyticum* and *Clostridium kluyveri* was designed to have metabolic complementarity and cooperative division of labor to facilitate lignocellulose degradation, resulting in a 50% increase in C6 carboxylate yield compared to individual strains [9, 14]. Similarly, metagenomics data from soil microbiome was analyzed using random forest (RF) and co‐occurrence network models to identify keystone species significantly correlated with herbicides residue, which were then isolated, genome sequenced and simplified to construct a SynCom to efficiently degrade eight herbicides [15].

In contrast to the “top‐down,” the “bottom‐up” strategy enables the potential of precise functional and ecological control. However, it is constrained by the availability of existing strain collections and databases [16], and it may result in the loss of naturally‐evolved interaction networks that could promote stability. To balance functional efficiency with ecological stability, recent studies have explored the integration of bottom‐up and top‐down strategies [17]. This involves merging enrichment strategies with functionally‐identified strains, utilizing process‐based models (simulating ecosystem dynamics and mass balance) alongside metabolic models (predicting metabolite fluxes and conversions) to optimize consortium design. For example, a Domestication‐Modeling‐Validation (DMV) pipeline leveraging environmental selection and key metabolic network analyses, can efficiently simulate and identify optimal SynCom configurations [18, 19]. Xu et al. utilized selective media to obtain functionally specific microbial communities and applied genome‐scale metabolic modelling to predict the community growth, metabolic profiles, and phenotypic traits. This approach enabled the determination of an optimal microbial combination that enhanced the degradation efficiency of atrazine [20].

### 1.4 Core Technologies

Microbial isolation and cultivation are essential, especially for “bottom up” SynCom construction (Table 3). Traditional methods rely on selective media and culture conditions optimized to isolate specific species or functional phenotypes from complex environments like soil or a plant rhizosphere [21, 22, 23, 24, 25]. The core challenge is the difficulty of finding cultivation conditions for the majority of microbial life, which results in a limited selection pool of strains to work with [26]. To address this issue, high‐throughput automated culture platforms are emerging. Techniques such as microfluidic chip‐based single‐cell isolation and cultivation significantly enhance strain isolation efficiency [3, 27]. High‐throughput pure‐culture platforms, integrated with bioinformatics, accelerate strain isolation through multi‐well plate cultivation, which allows parallel growth and reverse screening of cultures to identify strains with desired phenotypes [27, 28]. Microbial co‐cultivation technology is crucial for uncovering inter‐strain interactions [29]. However, while valuable, all‐pairwise screening (i.e., testing all possible combinations of strains), can become impractical as strain numbers increase (e.g., 27 strains yield 20,826 possible pairwise combinations). To overcome this bottleneck, one SynCom was constructed using a tiered screening strategy that first assessed paired cultures and prioritized interactions associated with significant phenotypic outcomes [22, 28, 29, 30, 31].

**TABLE 3:** Core technology.

Typology	Technology	Cases	Functionality	References
Microbial isolation and cultivation	Traditional separation and screening	Selective media and culture condition optimization	Over 90% environmental microorganisms are difficult to cultivate under laboratory conditions	[21, 22, 23, 24, 25, 26, 27]
	High‐throughput pure culture technology	Multi‐well plate cultivation	Reverse screening of results to identify desired strains	[28, 29, 30, 31]
SynComs design	Metabolomics‐driven community optimization	Substrate utilization and product synthesis	Cross‐feeding between members to improve functional stability	[32, 33]
	Model predictions	Genome‐scale metabolic models (GEMs)	Cooperative communities exhibit the opposite characteristics	[34, 35]
	Artificial intelligence (AI)	Automated cloud laboratories	Rapidly and accurately screen high‐potential SynCom combinations via deep learning	[3, 36]
SynComs optimization	Gene editing technology	Superbugs	Introduce desired metabolic or biosynthetic functions	[37, 38, 39]
	Biosensors	“Transmitter‐receiver” SynCom biosensor	Environment‐responsive regulation	[40]
	Encapsulation and immobilization techniques	Sodium alginate gel encapsulation technology	Regulate the stability of community interactions	[40]

Metabolomics‐driven community optimization focuses on substrate utilization and product synthesis, leveraging the distinct metabolic functions of different bacteria to form stable symbiotic communities [32]. For example, metabolomics of wastewater being treated to remove phenol and formaldehyde revealed that the bacterial community enriched during the gas‐phase domestication stage not only included *Pseudomonas putida*, which can complete the first step of degrading phenol and formaldehyde, but also a large number of polyhydroxybutyrate (PHB)‐accumulating bacteria, which can converted toxic intermediate carbon (C) sources into the PHB polymer. The engineered SynCom, based on this design, could degrade phenol and formaldehyde with 8‐fold higher efficiency than conventional methods [33].

Genome‐scale metabolic models (GEMs) are computational biology models that systematically reconstruct the complete metabolic network of an organism based on its whole‐genome annotation information. It integrates all known biochemical reactions within the organism (including enzymes, metabolites, transport proteins, and their stoichiometric relationships) and simulates the organism's metabolic capabilities and physiological functions in mathematical form (usually as a stoichiometric matrix). The models use rate kinetics for metabolic pathways to predict the growth dynamics of a species. Consequently, this can be used to predict metabolic interactions between species [34, 35]. In addition, AI‐integrated modeling strategies are emerging as powerful new tools for SynCom design [3]. These approaches combine computational simulation, algorithmic optimization, and multi‐omics data integration to rapidly and accurately identify high‐potential SynCom configurations using deep learning [3, 36]. However, the effectiveness of AI and machine‐learning models remains strongly dependent on the quality of input data and on subsequent experimental validation.

Gene editing technology is another tool for structure stabilization and function optimization, which allows precise, targeted modification of genomes to delete or introduce target metabolic or biosynthetic functions, enabling rapid creation of SynComs with specific capabilities [37, 38]. Strains can be genetically modified for targeted traits or functions, and then combined to construct SynComs with defined interactions. For example, in *Pseudomonas aeruginosa* MCCB 117 modification, researchers replaced the natural *phz* operon promoter with the strongly inducible promoter *pbad*, resulting in a 2.1‐fold increase in chloropyrifos production [39]. Importantly, strict risk assessment is mandatory before environmental release of genetically engineered microorganisms to prevent biological invasion or creation of “superbugs” [40].

## 2 Interaction Between SynCom members

### 2.1 Bacterial Interaction Can Facilitate Metabolic Flexibility

**FIGURE 2:**
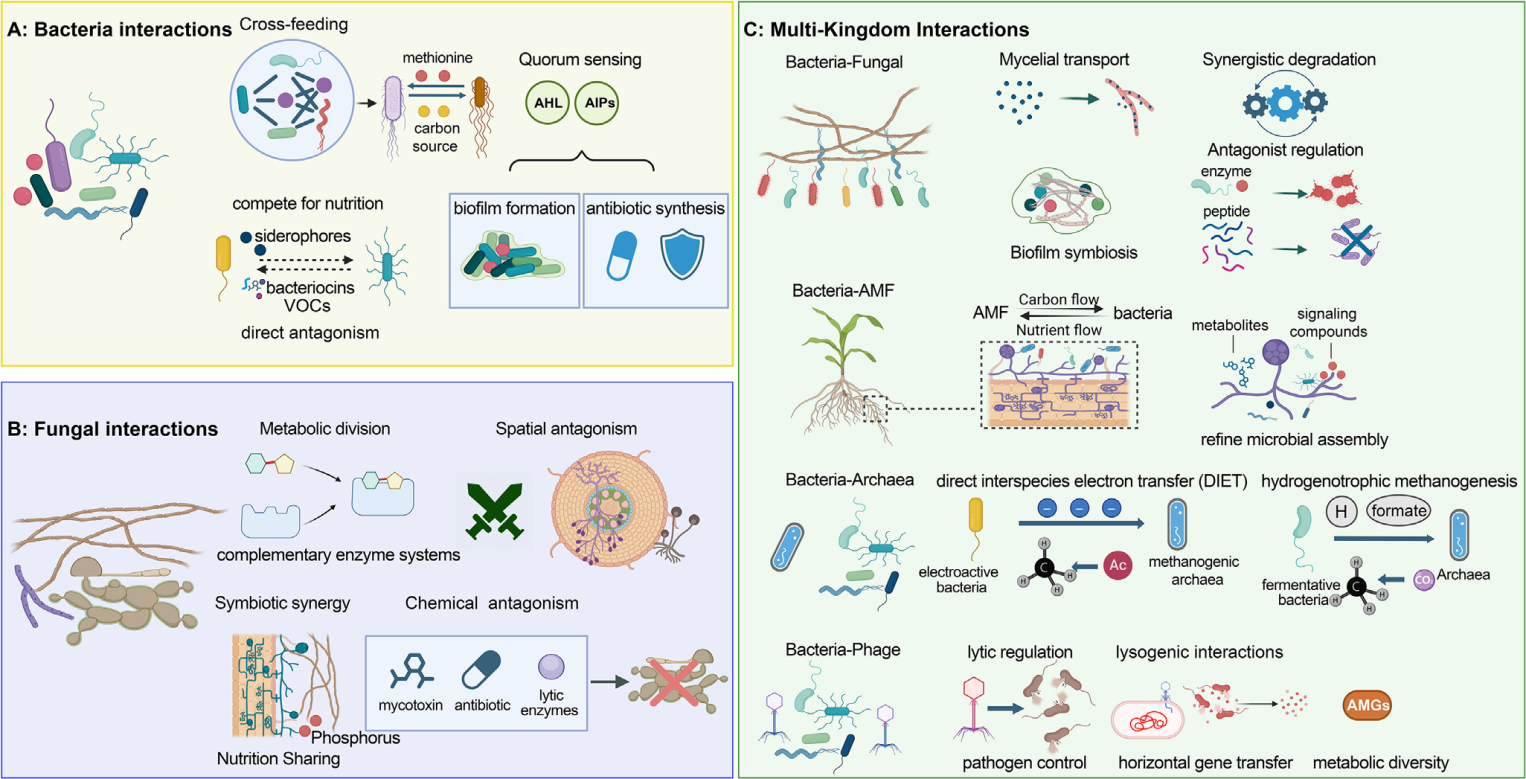
The interaction mechanisms among members in SynComs.

Bacterial interactions are governed in part by metabolic cross‐feeding and resource competition, which collectively shape the structure and function of a community (Figure 2) [41, 42]. Metabolic cross‐feeding involves the exchange of diverse metabolites, ranging from basic nutrients to complex biomolecules, and facilitates synergistic survival and proliferation [43]. This metabolic interdependence is ubiquitous in natural ecosystems. Auxotrophic bacteria, constrained by incomplete metabolic networks, rely on the supply of essential metabolites from other community members for survival [42]. For instance, *E. coli* and *Salmonella enterica* engage in reciprocal metabolic exchange, with *E. coli* acquiring methionine from *S. enterica* while providing a C source essential for the latter's growth [44]. Studies also report the communal sharing of critical growth factors, such as vitamin B_12_ (cobalamin), among community members [45]. Cross‐feeding not only promotes the sharing of nutrients but also enables functional specialization, particularly for degrading complex substrates. *Bifidobacterium* sp., for example, ferment complex carbohydrates to produce acetate and lactate, serving as C sources for other bacteria and enhancing overall community growth [46]. Consequently, cross‐feeding relationships significantly expand the collective metabolic niche of the community, enabling access to resources beyond the reach of individual members [46]. Distributing complex metabolic pathways across specialized strains circumvents metabolic bottlenecks and the accumulation of toxic intermediates within a single strain, while enabling modular assembly for specific functions [47]. An analysis of GEMs, covering most soil or aquatic environments, revealed that competitive communities, such as plant growth‐promoting rhizobacteria, have strong resistance to invasion but weak environmental adaptability, while cooperative communities, such as PHB‐producing bacterial communities, are the opposite, further highlighting the value of identifying the metabolic interactions that shape stability [34, 35].

Competition is a fundamental interspecies interaction that plays a critical role in shaping microbial community structure and function [43]. Bacteria employ a range of strategies to restrict the invasion and proliferation of competitors, including altering carbon source utilization preferences [48], secreting siderophores to capture limited iron resources [49], and forming biofilms to secure spatial dominance within ecological niches [50]. More direct antagonistic mechanisms include the production of bacteriocins by species such as *Bacillus* sp., which inhibit the growth of other Gram‐positive bacteria [51]. *Bacillus amyloliquefaciens*, for example, produces volatile organic compounds (VOCs) that not only suppress pathogens like *Ralstonia solanacearum* but can also drive the evolutionary attenuation of pathogen virulence [52]. Importantly, competitive and cooperative interactions among bacteria are not static but can shift dynamically. For instance, following the lysis of *E. coli*, *Salmonella* sp. exploits the released β‐galactosidase as a carbon source, thereby transforming an antagonistic interaction into a form of nutrient acquisition [53].

Complex mutualistic networks among bacteria are intricately regulated by quorum‐sensing (QS) systems. QS comprises diffusible chemical signaling molecules such as N‐acyl homoserine lactones (AHLs) [54] and autoinducing peptides [55] to coordinate cooperative and competitive behaviors within populations. QS mediates processes like antibiotic synthesis and biofilm formation to enhance communal defense, while also enabling dynamic adaptation of response networks to environmental fluctuations [56]. Crucially, QS also facilitates complex inter‐kingdom signaling with a host. Plants can perceive bacterial AHL signals, which can induce systemic resistance and enhance disease tolerance [54]. The intensity of bacterial interactions is modulated by the synergistic influence of intrinsic and extrinsic factors. For example, extrinsic factors like temperature can profoundly impact interaction dynamics by regulating microbial metabolic activity, community structure, and functional gene expression [57]. Similarly, nutrient availability can significantly alter the frequency of negative interactions within coexistence networks, serving as a direct indicator that resource abundance governs the intensity of competition. Under resource‐sufficient conditions, competitive pressure is alleviated; conversely, resource limitation drives native communities to fortify resilience by occupying ecological niches [58]. Intrinsically, the community's compositional structure and metabolism govern interaction mechanisms. Niche differentiation and adaptive divergence can underpin species coexistence, while processes such as metabolite exchange, QS signal, and host immune feedback collectively weave a complex interaction network at the micro‐scale [59].

### 2.2 Fungal Metabolism can Support Structural Stability

The functional dynamics of fungal ecosystems are primarily driven by competition and synergism. Synergistic interactions arise from the mutualistic sharing of resources among different fungi, which also results in metabolic division of labor, whereby one fungal species secretes complementary enzyme systems enabling the cascade degradation of complex substrates to support other fungal species [60, 61]. For example, during straw decomposition, interactions between *Phanerochaete chrysosporium* and *Trichoderma reesei* significantly enhance the degradation efficiency of lignin and cellulose [62]. Similarly, in petroleum hydrocarbon bioremediation, *Aspergillus* sp. can degrade alkanes, while *Penicillium* sp. can degrade aromatic hydrocarbons, thereby improving remediation potential [63]. Symbiotic facilitation represents another important type of synergy. The hyphal interaction networks formed by arbuscular mycorrhizal fungi (AMF), for example, facilitate cross‐host nutrient sharing and significantly enhance phosphorus (P) uptake and stress tolerance of plants [64]. Harnessing such symbiotic relationships can contribute to increased crop yields and improved ecological outcomes.

Competition manifests as both spatial and chemical interference. For example, fast‐growing fungi like *Trichoderma* sp. can rapidly occupy ecological niches, leading to hyphal avoidance or zonal colonization by other fungi [65, 66]. *Trichoderma harzianum*, for example, effectively suppresses the pathogen *Fusarium* sp. through rapid root colonization [67]. Chemical antagonism involves fungi suppressing competitors by secreting antimicrobial compounds such as antibiotics, mycotoxins, or lytic enzymes. *Trichoderma* sp. inhibits *Rhizoctonia sclerotia* by producing metabolites like viridin, which can reduce reliance on chemical fungicides and maintain soil health [68]. Physiologically, rapid colonizers (e.g., *Trichoderma*) dominate early niche occupation through first‐come, first‐serve competitive advantage [66], while slower‐growing fungi (e.g., *Phanerochaete*) achieve efficient resource conversion via synergistic metabolic division of labor [62]. Environmentally, resource heterogeneity, such as carbon/nitrogen (C/N) ratio, modulates the competitive‐synergistic dynamic equilibrium, and community complexity buffers interaction conflicts through functional redundancy [69]. This coordination and functional division among different fungal members within communities ensures the stability of fungal SynComs and their continued desired phenotypic efficacy.

### 2.3 Cross‐Kingdom Interactions in SynComs

Early SynCom designs primarily focused on bacterial taxa such as *Clostridium* and *Pseudomonas, or fungal consortia as identified in the previous section*. Contemporary frameworks, however, increasingly emphasize multi‐kingdom consortia incorporating fungi, archaea, and viruses to more accurately reproduce ecological complexity and functional interdependence [70, 71].

#### 2.3.1 Bacteria‐Fungi Interactions: Synergy and Antagonism

Fungal hyphae act as transport networks, enabling bacterial movement across soil matrices. For example, *Mortierella* sp. LEJ702 facilitates the migration of *Arthrobacter globiformis* D47, expanding the spatial range of herbicide degradation [72]. Similarly, *Penicillium* hyphae enhance the dispersal of *Pseudomonas aeruginosa* by 1.4‐fold, increasing bacterial functional diversity [73]. Cross‐kingdom metabolic cooperation often underpins the degradation of complex substrates. During polycyclic aromatic hydrocarbon (PAH) degradation, fungi initiate oxidation of high‐molecular‐weight or insoluble compounds, converting them into low‐molecular‐weight, soluble intermediates that serve as substrates for bacteria [74]. Fungal exudates further supply carbon sources that stimulate bacterial growth and pollutant degradation [75]. In the rhizosphere, biofilm‐mediated associations exemplify mutualistic interactions, whereby bacteria colonize fungal surfaces to access nutrients, while fungal networks guide bacterial migration toward nutrient‐rich zones such as plant root surfaces. These cooperative interactions enhance substrate degradation via metabolic complementarity. Notably, *Pseudomonas aeruginosa* often forms tripartite biofilms with yeasts and filamentous fungi on plant surfaces, demonstrating biofilm architecture as a strategy for defense and resource sharing in the phyllosphere [76].

Conversely, antagonistic interactions also contribute to ecosystem resilience. Bacterial chitinases can lyse fungal cell walls (e.g., engineered *Phomopsis liquidambaris* targeting *Fusarium graminearum*) [77], while fungal antimicrobial peptides suppress bacterial proliferation [78]. Resource competition, e.g., for iron or carbon, selects for the most competitive taxa. These relationships are context‐dependent, i.e., at low concentrations, bacterial QS signals promote biofilm symbiosis [79], whereas high concentrations inhibit fungal sporulation [80]. Fungi can disrupt bacterial QS via VOCs; for instance, engineered yeast expressing AHL‐lactonase suppresses *Botrytis* virulence [81]. Targeted parasitism, such as *Bdellovibrio* preying intracellularly on *Pythium*, illustrates how cross‐kingdom antagonism maintains microbial equilibrium under environmental stress [82].

#### 2.3.2 Bacteria‐AMF Interactions

AMF are fundamental components of the plant microbiome and key mediators of terrestrial nutrient fluxes. Through symbiosis with plants, AMF exchange phosphorus (P) and nitrogen (N) for host‐derived photoassimilates such as lipids and sugars [83]. Bacteria participate actively in this tripartite association, with some AMF species even harboring intracellular endobacteria. Extraradical hyphae (ERH) create hyphospheric niches where bacterial communities utilize fungal‐transferred, plant‐derived carbon exudates [84]. Bacterial extracellular enzymes complement AMF's limited capacity for organic nutrient mineralization, enabling the depolymerization of complex substrates [85].

AMF also influence microbial community assembly through chemical signaling. They secrete metabolites and signaling molecules that selectively recruit bacterial partners to the hyphosphere [86]. In turn, plant receptors for chitin‐oligosaccharides (COs), such as those in *Medicago truncatula*, regulate root‐associated bacterial communities, while CO‐producing bacteria stimulate plant symbiotic signaling [87]. AMF networks further act as transport conduits, mobilizing phosphate‐solubilizing bacteria toward organic P reservoirs, thereby accelerating mineralization and supporting bacterial colonization [88]. Reciprocal bacterial contributions via nitrogen supply and phytohormone modulation can enhance AMF vitality, establishing a self‐sustaining nutrient exchange system that maximizes resource partitioning efficiency in engineered SynComs.

#### 2.3.3 Bacteria‐Archaea Interactions

Syntrophic interactions between bacteria and archaea are central to anaerobic energy metabolism, operating through mechanisms such as direct interspecies electron transfer (DIET) and hydrogenotrophic methanogenesis. In DIET, electroactive bacteria (e.g., *Geobacter metallireducens*) transfer electrons directly to methanogenic archaea (e.g., *Methanosaeta harundinacea*), accelerating acetate conversion to methane [89]. In hydrogen‐based syntrophy, fermentative bacteria such as *Syntrophobacter fumaroxidans* produce H_2_ or formate that is consumed by *Methanospirillum hungatei* to reduce CO_2_ to CH_4_, maintaining energy fluxes within the consortium [90]. A similar hydrogen transfer occurs between *Desulfovibrio* sp. and *Methanobrevibacter arboriphilus* AZ [91].

Archaea like *Nitrososphaera* also contribute to carbon cycling in extreme environments [92], and such interactions have been incorporated into engineered SynComs. For example, a consortium combining the carbon‐sequestering archaeon *Nitrososphaera viennensis* EN76 with the phenanthrene‐degrading bacterium *Sphingobium* sp. RS2 increased community modularity and robustness in phenanthrene‐contaminated soils, improving resistance to pollutant stress [93]. More broadly, networks of ammonia‐oxidizing archaea (AOA), ammonia‐oxidizing bacteria (AOB), complete ammonia oxidizers (comammox), and nitrite‐oxidizing bacteria (NOB) collectively mediate nitrification—a pivotal process in the global nitrogen cycle [80].

#### 2.3.4 Bacteria‐Phage Interactions: Regulators of Microbial Equilibrium

Bacteriophages serve as key regulators of soil microbial communities, influencing ecosystem processes and enhancing plant stress tolerance [94]. Environmental stressors drive phage–host co‐evolution, sometimes converting parasitic relationships into mutualistic adaptations that confer resilience in extreme environments [95]. In their lytic mode, phages directly suppress pathogens: for example, the P2‐like phage φRSY1 lyses *Ralstonia solanacearum* and reduces its virulence by inducing hypermotility and aggregation [96]. Giant phages such as *Agrobacterium* phage Atu_ph07 and *Xanthomonas* phage XacN1 selectively target phytopathogens responsible for crown gall and citrus canker [97]. In lysogenic associations, phages enhance bacterial fitness via horizontal gene transfer and auxiliary metabolic genes (AMGs), for instance, *phoH*‐mediated phosphorus tolerance [98] or *Pf1*‐associated biofilm resistance in *Pseudomonas aeruginosa* [99]. Phage consortia thus play a crucial role in maintaining soil microbial homeostasis by exerting top‐down pathogen control, reducing competition among beneficial bacteria, and preserving metabolic diversity under stress. Together, these processes lower disease incidence and enable precision biocontrol in plant–microbe systems [100].

## 3 SynComs Metabolically Interact With Endogenous Communities

### 3.1 SynComs Shift Native Microbial Community Diversity

**FIGURE 3:**
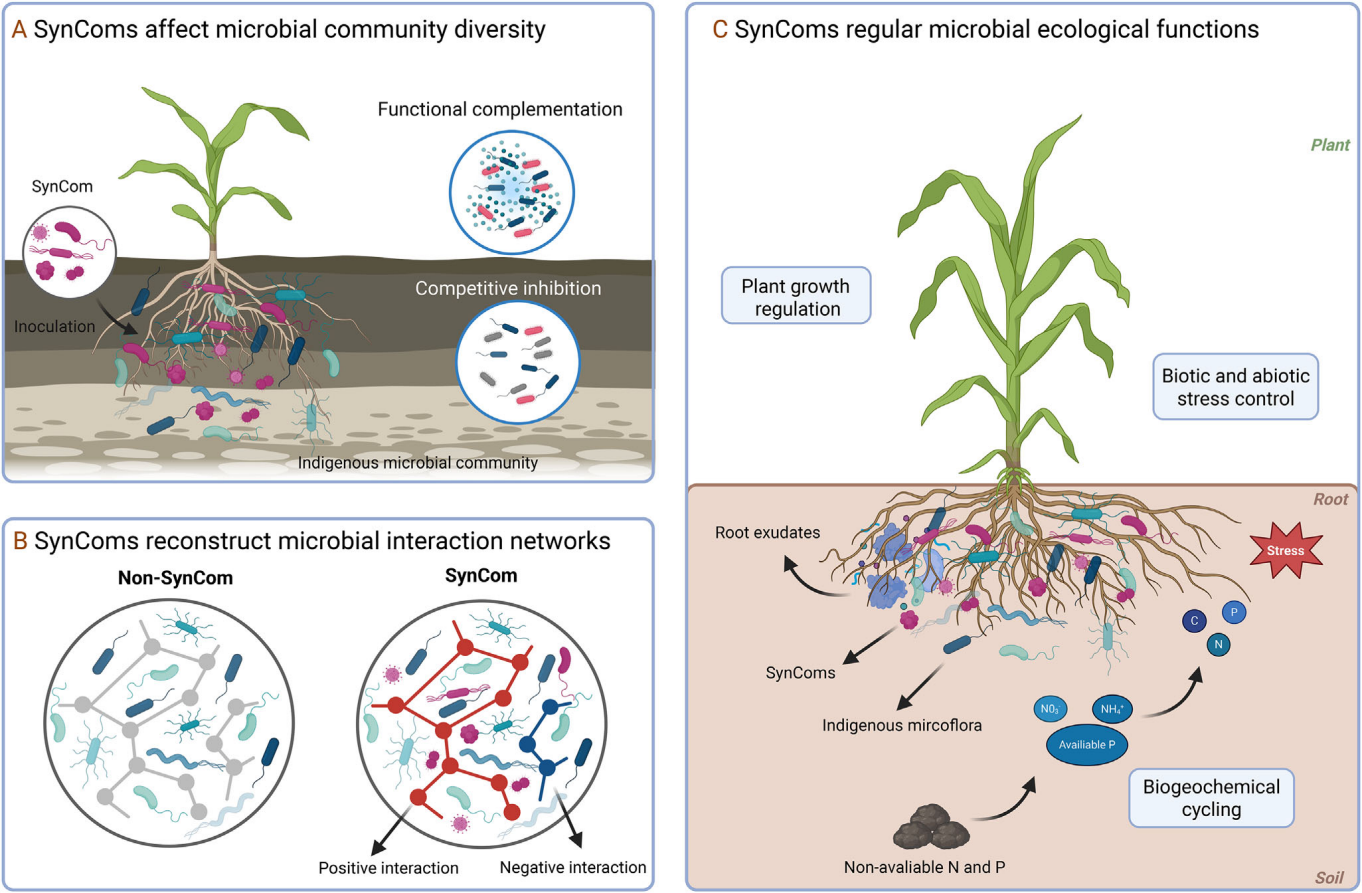
Mechanisms of SynComs in shaping plant‐microbe‐soil interactions.

SynComs introduced into soil have been shown to directly influence the diversity and composition of soil or plant‐associated microbial communities. For example, in degraded arid soils in Northwest China, the addition of a SynCom led to an increase in both the abundance of specific bacterial species and overall bacterial diversity, accompanied by an increase in microbial ecological stability [101]. SynComs can complement degraded ecosystems by introducing functional microbial taxa that occupy vacant ecological niches and restore community balance [102]. Through improved regulation of resource distribution, they can also mitigate competition among native microorganisms and prevent the exclusion of less‐adaptive species. Moreover, SynCom members often secrete extracellular polymeric substances (EPS) and plant growth–promoting metabolites that enhance soil microhabitat conditions, such as water retention and nutrient availability, thereby stimulating the recovery and activity of dormant endogenous microbes [103]. Additionally, a SynCom introduced into the *roots of Astragalus membranaceus* enhanced root biomass and suppressed root rot in part by modulating the endogenous microbial community [104]. In the pepper rhizosphere, SynCom application significantly increased microbial diversity and altered community composition, indicating that the introduced communities could indirectly optimize endogenous community structure by modifying root exudate composition or by recruiting native beneficial bacteria through resource competition [105]. Further evidence comes from a continuous apple cultivation experiment, where a SynCom comprising 8 species increased bacterial species richness and elevated the relative abundance of potentially beneficial bacteria [106]. Recent studies in agricultural ecosystems have highlighted the targeted effects of functional SynComs. For instance, N‐fixing SynComs (e.g., combinations of *Rhizobium* sp. and *Glomus* sp.) not only supplement N input but also upregulate the expression of N metabolism genes in native microbes, thereby promoting the coexistence of diverse functional groups [107]. Also, AMF‐based SynComs enhance the connectivity between native microbial taxa by extending hyphal networks, facilitating nutrient exchange and microbial colonization [108]. These findings indicate SynCom application can change the endogenous microbial community in multiple agricultural settings. SynComs may enhance microbial diversity and increase ecosystem stability, potentially by introducing new functional metabolism and/or stimulating the growth of endogenous microbes whose metabolisms result in changes in the endogenous community structure [109]. Maintaining or increasing community diversity is often considered a positive indicator, as the introduction of an exogenous beneficial community can stimulate adaptive responses from native endogenous species (Figure 3A). Ultimately, SynComs may contribute to the formation of a more plant‐friendly microbial community structure.

While the application of a SynCom can enhance microbial community diversity, this outcome is not guaranteed. In some cases, SynComs may trigger competitive exclusion, leading to a decline in the diversity of certain species [110]. This is indicative of the fact that we do not yet fully understand all the dynamic processes between various environmental factors, local microbial communities, and SynCom components, and we may never know everything. Studies highlight the need for caution when assembling rhizosphere SynComs, particularly in avoiding disproportionate amplification of a single functional group, as this may lead to the competitive exclusion of other ecologically important taxa. For example, an overabundance of *Rhizobium sp*. could suppress native phosphate‐solubilizing *Azospirillum sp*., thereby disrupting nutrient cycling dynamics [110]. Therefore, to maintain the ecosystem function, SynCom design must prioritize functional complementarity both within the membership and with the endogenous community into which it is introduced, while dosage must be configured to support constituent microbes. Furthermore, environmental factors such as the richness of the pre‐existing community and soil nutrient status will regulate the survival and interactions of SynComs, thereby influencing their success. However, while these factors must be considered during the SynCom application, and data that might inform reliable implementation across diverse systems remains limited.

### 3.2 SynComs Drive Endogenous Microbial Metabolic Network Structure

The introduction of SynComs into non‐sterile soil environments often reshapes microbial interaction networks, altering both positive (mutualism, commensalism) and negative (competition, and antagonism) associations among community members. These changes ultimately influence the structure and function of the entire ecological network [111].

For any exogenous microorganism to successfully establish within an endogenous community, it must first overcome competitive pressures from native microbes. Extensive research and field observations demonstrate that introduced strains often fail to colonize effectively unless they can compete for limited resources, including nutrients, space, and host colonization sites, or produce inhibitory metabolites that suppress resident microorganisms [110, 112]. However, SynComs can also foster positive synergies with endogenous communities. For instance, they may provide novel metabolites or nutrients to stimulate endogenous microbial growth, or collectively enhance plant‐beneficial functions, which may reciprocally influence endogenous microbes. Studies indicate that SynComs can selectively promote the proliferation of specific endogenous beneficial fungi and bacteria, further amplifying plant growth improvement [105].

Through these dynamic interactions, SynComs can reshape the architecture of microbial interaction networks, typically increasing their complexity, modularity, and average connectivity (Figure 3B). Enhanced modularity often indicates the formation of tightly linked, interconnected functional subgroups within the community [113]. SynComs may serve as novel network hubs, bridging specific native microbes and facilitating more efficient functional specialization. Additionally, the introduction shifts the assembly processes of rhizosphere microbial communities from stochastic toward more deterministic patterns, reflecting their ability to promote more stable and predictable community structure [105, 106, 111, 113]. Notably, SynComs may in some cases assume central roles within these networks, acting as new interaction cores that rebalance microbial dynamics, thereby suppressing pathogens (via negative interactions) while enriching mutualistic taxa (via positive interactions).

While most SynCom research has focused on bacterial interactions, emerging studies explore synthetic fungal communities or bacterial‐fungal consortia [110, 112]. Although limited, existing evidence suggests that cross‐kingdom interactions hold greater potential for enhancing network robustness, particularly when SynComs include substantial metabolic and taxonomic diversity. For example, in systems co‐inoculated with AMF and bacteria, the fungal hyphae provide a physical network for bacterial dispersal, while bacteria modulate fungal community assembly [110]. Similarly, in SynComs designed to suppress tomato pathogens, bacteria directly antagonized pathogenic fungi, whereas fungi hosted symbiotic bacterial species crucial for sustaining plant immunity [114]. Recent advances in agricultural ecology have revealed that cross‐kingdom SynComs (e.g., combinations of N‐fixing bacteria, AMF, and phosphate‐solubilizing fungi) form “functional modules” that synergistically regulate nutrient metabolism. AMF hyphae transport P to plants and bacteria, N‐fixing bacteria provide ammonium to fungi, and phosphate‐solubilizing fungi release bound P in soil, creating a closed nutrient cycle that enhances network stability [115]. In saline‐alkali soils, bacterial‐AMF SynComs upregulate the expression of related salt‐tolerant genes in plants, forming a metabolic alliance that improves stress resistance [116, 117]. As our understanding of cross‐kingdom microbial interaction networks advances, the rational design of targeted synthetic micro‐ecosystems—encompassing bacteria, fungi, archaea, and even algae‐ will likely improve the stability and functionality of SynComs at the network level.

### 3.3 SynComs Enhance Ecological Functionality

The interactions between exogenous SynComs and endogenous microbes extend beyond micro‐scale community restructuring, generating cascading effects that influence macro‐scale ecological functions and host performance [118]. These indirect impacts encompass improvements in plant growth and health, modulation of soil nutrient cycling and environmental conditions, and enhanced disease control (Figure 3C). Introduced SynCom members may function as novel hub nodes within microbial networks, triggering community‐level cascades. As network hubs, SynComs can stimulate beneficial microbe proliferation while indirectly suppressing pathogens, establishing a self‐reinforcing cycle of positive ecological feedback. For instance, introducing a plant‐growth‐promoting SynCom into the apple rhizosphere significantly increased the abundance of *Pseudomonas sp*. and enriched biofilm‐related genes, which then decreased host disease incidence [106]. Conversely, negative effects may emerge upon the removal or dysregulation of an endogenous hub microbe. The removal of a critical rhizosphere species has been shown to trigger the near‐complete collapse of the symbiotic communities [119]. Similarly, overcompetitive SynComs or those producing broad‐spectrum antagonistic metabolites may inadvertently suppress multiple symbiotic microbes, leading to functional decline in the community [120, 121].

The interactions of SynCom‐endogenous microbes can also profoundly influence biogeochemical cycles. Functionally specialized SynComs can modify the transformation dynamics of C, N, and P, thereby affecting soil fertility and environmental sustainability. In desert soil restoration studies, SynCom application enhanced soil nutrient cycling and reshaped the relationship between soil organic matter (SOM) and microbial diversity. Compared to chemical fertilization alone, SynCom treatment yielded significantly greater increases in soil available N, P, K, and other nutrients, while positively influencing soil C sequestration capacity [101]. Recent studies on agricultural ecosystems have demonstrated that N‐fixing SynComs increase soil N content by enhancing nitrogenase activity, thereby achieving efficient conversion and utilization of soil nutrients;[122] phosphate‐solubilizing SynComs can convert unavailable P (e.g., calcium phosphate, iron phosphate, and aluminum phosphate) into plant‐available forms through acidification, chelation, exchange reactions, and polymer formation;[123] carbon‐fixing microbial communities mainly input C through two forms, self‐fixation of C and microbial‐mediated input of organic C into plants. Xiao et al. [125] confirmed the key role of autotrophic microbial communities in paddy field ecosystems in fixing CO_2_ and increasing the accumulation of organic C pools [124]. Collectively, SynComs can generate positive cascading effects on soil nutrient cycling and fertility through multiple pathways, such as N‐fixing bacteria increasing soil N, P‐solubilizing bacteria enhancing P availability, and plant‐growth‐promoting bacteria (PGPB) stimulating plant growth and therefore greater belowground biomass that stores C.

Beyond their role in nutrient cycling, SynComs can also enhance plant resilience to biotic and abiotic stresses, thereby influencing ecosystem stability. These effects are often mediated through intimate interactions with plant roots. SynComs modulate root exudate composition, for example, increasing the secretion of proline and soluble sugars to improve osmotic regulation under drought or salinity stress [125]. By colonizing root surfaces and forming biofilms, SynCom members reduce water loss and restrict the uptake of toxic ions, such as Na⁺ in saline soils [113]. They can also activate plant stress‐response pathways, including the induction of drought‐ and salt‐tolerance genes (e.g., *DREB1D*, *SOS1*) and the upregulation of antioxidant enzymes such as superoxide dismutase and peroxidase, which mitigate oxidative damage caused by extreme environmental conditions [113, 126].

Synergistic associations between plant growth‐promoting bacteria (PGPB) and AMF further amplify these protective effects. Co‐inoculation has been shown to enhance water‐use efficiency and alleviate drought stress in maize and wheat [127, 128]. In semi‐arid field trials, a drought‐tolerant SynCom composed of *Bacillus subtilis* and *Acaulospora laevis* increased maize yield by reducing oxidative stress, improving soil water retention via extracellular polymeric substance (EPS) secretion, and protecting chlorophyll content [103, 129]. In addition to abiotic stress tolerance, certain SynComs stimulate induced systemic resistance (ISR), enhancing plant defenses against pathogens and heavy metals through coordinated microbial and host signaling [130]. Improvements in plant health, in turn, reshape rhizosphere microbial diversity and function, establishing a positive feedback loop that reinforces soil and ecosystem stability. Collectively, these interactions illustrate how SynComs can simultaneously boost crop productivity, sustain soil function, and strengthen ecosystem resilience under environmental stress.

## 4 Functionality and Application Scenarios

### 4.1 Soil Constraint Mitigation and Fertility Improvement

**FIGURE 4:**
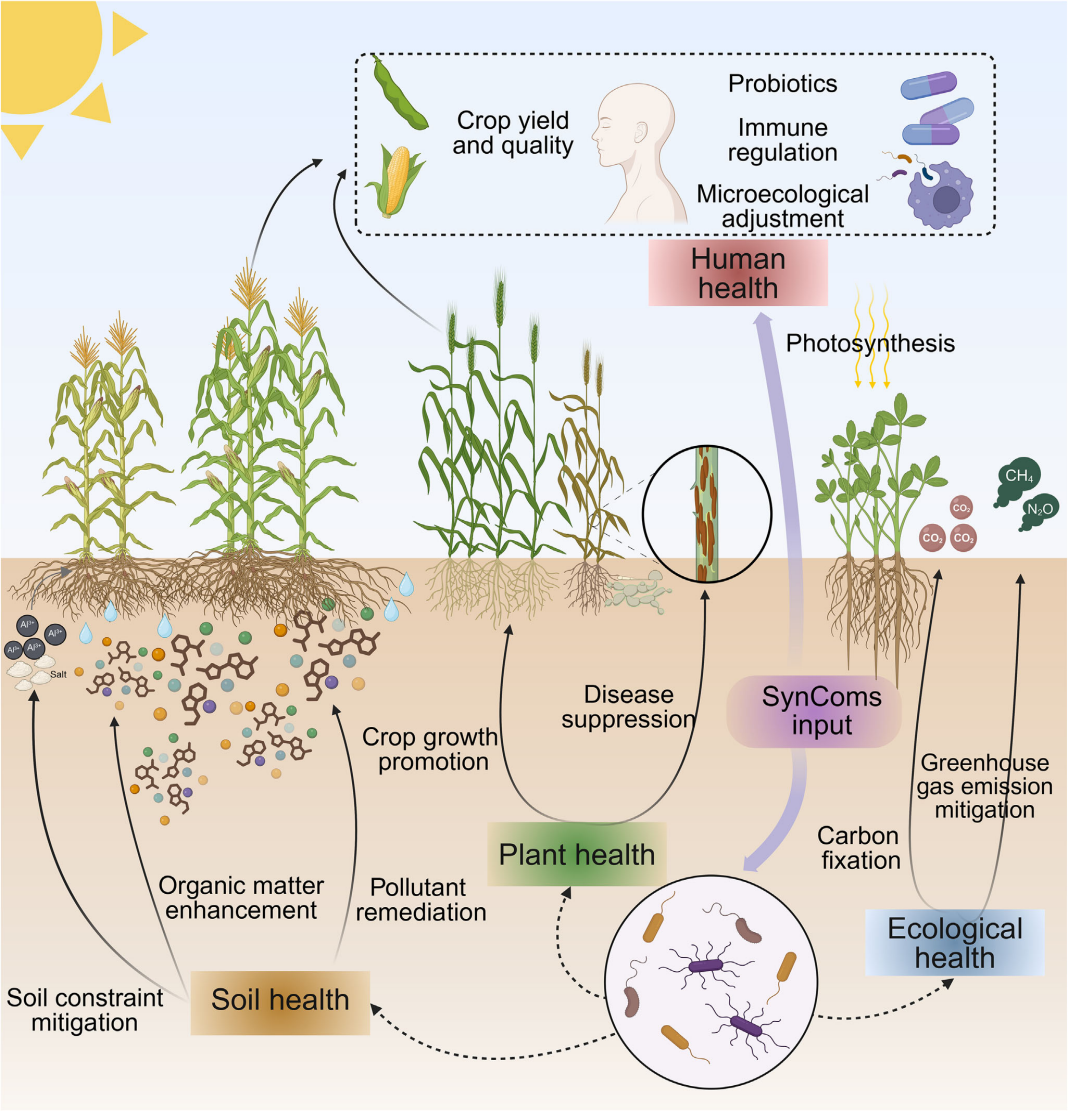
The application scenarios model and regulatory mechanisms for SynComs.

Abiotic stresses, including drought, soil acidification, and salinization, pose severe threats to global agricultural productivity and ecosystem stability, which degrade soil health, reduce crop yields, and thereby exacerbate the food security crises (Figure 4, Table 4) [131]. In recent years, SynComs have emerged as a promising approach to tackle this problem, demonstrating significant potential in mitigating constraint factors, enhancing plant stress resilience, and promoting agricultural production [132]. For example, high concentrations of Na⁺ in saline soils cause combined damage through osmotic stress, ion toxicity, and oxidative injury to plants [133]. SynComs developed from mangrove plant leaves, comprising *Pantoea stewartia* A and *Bacillus marisflavi* Y25, exhibit functional complementarity by producing salt‐stress‐mitigating metabolites such as L‐lysine, L‐glutamic acid, aspartic acid, and betaine. Inoculation onto rice significantly up‐regulated the expression of key genes involved in Na⁺ transport (*OsSOS1*), auxin efflux (*OsPIN1*), and stress regulation (*OsCIPK15*), effectively reducing the negative impacts of salt stress [134]. Research found that inoculation with a SynCom, composed of EPS‐producing *Streptomyces* sp. AB‐11 and *Pseudomonas* sp. BC‐II‐20, increased the membrane stability index and decreased the electrolyte leakage index by 16% in *Triticum aestivum* under 200 mmol/L NaCl stress [135]. A microalgae‐bacteria SynCom containing *Azotobacter beijerinckii* and *Chlorella pyrenoidosa* decreased soil pH by 5.5% under saline‐alkaline stress [136]. Soluble aluminum (Al^3^⁺) in acidic soils severely damages plant roots, inhibiting cell division, elongation, and membrane polarization, while concurrently inducing mineral nutrient deficiencies and suppressing plant growth [137]. A highly efficient Al^3^⁺‐tolerant SynCom composed of *Pseudomonas* sp. D95 and *Rhodococcus* sp. D96 promoted P uptake from the topsoil layer in rice, increasing plant height by 4.15%, leaf chlorophyll content by 9.81%, and grain yield by 26.36% [138]. Furthermore, global changes are increasing the risk of soil drought, imposing limitations on water and nutrient acquisition for crop growth. Studies have shown that a drought‐tolerant bacterial SynCom, comprising *Bacillus subtilis*‐FAB1 and *Pseudomonas azotoformans*‐FAP3, promoted wheat growth under drought stress, evidenced by increases in plant height (8.4%), fresh weight (21.2%), grain yield (14.1%), and straw yield (22.2%) [139]. Drought also induces iron deficiency symptoms in crops. A bacterial‐fungal SynCom comprising *Pseudomonas* sp. RU47, *Bacillus atrophaeus* ABi03, and *Trichoderma harzianum* OMG16 modulated phytohormone balance and stimulated the root release of the iron‐chelating metabolite benzoxazinoids [140]. A recent study indicated that incorporating species specifically screened under particular stresses enhances the regulatory efficacy of SynComs under those specific conditions [141].

**TABLE 4:** Example studies of the functions and application scenarios of SynComs.

Application scenario	SynComs	Functionality	References
**Soil constraint mitigation**	*Pantoea stewartii* A and Bacillus marisflavi Y25	Salt and alkali tolerance	[134]
	*Streptomyces* sp. and Pseudomonas sp.		[135]
	*Azotobacter* beijerinckii and Chlorella pyrenoidosa		[136]
	*Pseudomonas* sp. D95 and Rhodococcus sp. D96	Acid tolerance	[138]
	*Bacillus subtilis*‐FAB1 and Pseudomonas azotoformans‐FAP3	Drought tolerance	[139]
**Soil fertility improvement**	*Bacillus* subtilis and Bradyrhizobium japonicum	SOM enhancement	[142]
	*Bacillus* niacini, Agrobacterium tumefaciens, Klebsiella pneumoniae, *P*seudomonas furukawaii and Klebsiella variicola		[143]
	Lactic acid bacteria, yeast and Bacillus		[144]
	*Azotobacter* sp., Klebsiella sp., Bacillus sp., Polycyclovorans sp., Ramlibacter sp. Nocardioides sp.		[101]
	*Bacillus cereus*, Bjerkandera adusta, Trichoderma harzianum, Cladosporium cladosporioides and Cladosporium tenuissimum		[145]
	*Serratia* sp. and *Enterobacter* sp.		[146]
	*Rhizophagus intraradices* (BEG141) and *Funneliformis mosseae* (BEG12)		
**Crop productivity enhancement**	*A. xylosoxidans* Z2K8, Burkholderia sp. Z1AL11, K. variicola R3J3HD7, P. diazotrophicus Z2WL1, P. ananatis E2HD8 and P. protegens E1BL2.	Corn growth promotion	[150]
	*Rhizophagus irregularis* and *Devosia* sp. ZB163	*Prunella vulgaris* growth promotion	[151]
	*Pseudoduganella* sp. 1H4, Pseudoduganella sp. 9C5, Asticcacaulis sp. 9G4, Ensifer sp. 10D7, Massilia sp. 22G3 and Ensifer sp. 10C12	Arabidopsis growth promotion	[127]
	*Bacillus subtilis*, Trichoderma harzianum, Trichoderma asperellum and Aspergillus sp.	Pepper growth promotion	[152]
**Crop disease suppression**	*Trichoderma harzianum* OMG16 and Bacillus velezensis FZB42	Long‐spored yellow wilt disease inhibition	[155]
	*Stenotrophomonas* sp., Rhizobium sp., Ochrobacterium sp., and Advenella sp.	Astragalus root rot inhibition	[104]
	*Bacillus* and 10 other strains	Tomato wilt disease inhibition	[157]
	*Pseudoxanthomonas mexicana* and 16 other strains	Watermelon wilt disease inhibition	[32]
	*Chitinophaga* sp. and Flavobacterium sp.	Root rot disease inhibition	[158]
**Conventional pollutants remediation**	*Pseudomonas* sp. ADP and Arthrobacter sp.	Herbicide degradation	[159]
	*Enterobacter* sp. A11 and Comamonas sp. A23	Heavy metal degradation	[160]
	*Phlebia brevispora, Enterobacter* sp. TN3W‐14 and Pseudomonas sp. TN3W‐8	PAHs degradation	[161]
	*Acinetobacter baumannii* and Talaromyces sp.	Petroleum hydrocarbon degradation	[162]
	*Fomitopsis pinicola* and Ralstonia pickettii		[163]
	*Pseudomonas* sp. S1, Psychrobacter sp. S2, Arthrobacter sp. S3, and Bacillus sp. S4	Co‐degradation	[15]
	*Pseudomonas putida* C2 and Paenarthrobacter ureafaciens AT		[164]
**Emerging contaminants restoration**	*Rhodococcus jostii, Pseudomonas putida*, and two metabolically engineered Bacillus subtilis strains	Microplastic degradation	[168]
	*Glutamicibacter* sp. ZJUTW, Cupriavidus sp. LH1, and Gordonia sp. GZ‐YC7	PAEs degradation	[169]
**Microbial carbon fixation**	*Chlamydomonas reinhardtii* and Escherichia coli	Carbon sequestration enhancement	[170]
	hydrogen‐oxidizing/sulfur‐oxidizing bacteria and Geobacter sp.		[172]
	microalgal‐bacterial SynCom		[175]
**Microbial emission reduction**	Methylomonas sp. and Clostridium sp.	Methane emission reduction	[176]
	*Pseudomonas Fluorescens* and AMF	N_2_O emission reduction	[178]

SynComs can also enhance SOM content and restore soil fertility. For example, in agricultural systems, a SynCom comprising *Bacillus subtilis* and *Bradyrhizobium japonicum*, combined with organic amendments, synergistically increased soil dehydrogenase (DHA) and alkaline phosphatase (ALP) activities [142], consequently enhancing the transformation efficiency and pool size of soil available N, P, and K. Similarly, a SynCom was able to significantly increase soil available nutrient content, with alkaline hydrolysable N by 49.46%, available P by 99.51%, and available K by 19.38% [143]. SynComs can also drive SOM turnover and stabilization by boosting extracellular enzyme activity. Research suggested that a SynCom composed of plant growth‐promoting rhizobacteria (PGPRs) accelerated organic residue mineralization by enriching key genera involved in organic matter degradation, thereby promoting the activity of extracellular enzymes such as *β*‐glucosidase (BG), DHA, and ALP [101]. A SynCom can also significantly enhance BG (34.3–92.4%) and phenol oxidase (PHO, 49.3%–60.6%) activities by modulating soil microbial community structure and function, accelerating SOM mineralization and transformation [144]. Additionally, in composting applications, a SynCom composed of *Bacillus* sp. and *Trichoderma* sp., significantly accelerated lignocellulose degradation and promoted SOM humification by regulating the abundance of fungal community *Thermomyces sp. and Sordariomycetes* sp [145]. Furthermore, SynComs can facilitate the formation of stable aggregates through synergistic microbial‐EPS‐hyphal interactions [146]. The inoculation of AMF significantly enhances soil aggregate stability in calcareous soils through hyphal networks and glomalin, promoting erosion control [147]. This process not only increases SOM content but also enhances the persistence and resistance to decomposition of the soil C pool through improved aggregate structure, offering a biological pathway for soil C sequestration [132].

### 4.2 Crop Productivity Promotion and Disease Suppression

In the pursuit of sustainable modern agriculture, SynComs are emerging as versatile biological inoculants, providing systemic support for crop growth and disease suppression [148]. Research indicated that SynComs can directly improve plant uptake of nutrients such as N and P, enhancing nutrient acquisition capacity and efficiency [149]. For instance, a SynCom, comprising *Burkholderia* sp. Z1AL11, *Achromobacter xylosoxidans* Z2K8, *Burkholderia* sp. Z1AL11, *Kosakonia pseudosacchari* Z2WD1, *Kosakonia pseudosacchari* Z2WD1, *Pantoea ananatis* E2HD8, and *Pseudomonas protegens* E1BL2, leveraging complementary functions including N fixation, P solubilization, and auxin production, significantly increased maize growth [150]. Specifically, root length and dry weight were 3.3‐fold and 3‐fold higher than the control, respectively, while shoot length and dry weight reached 1.4‐and 2.3‐fold, respectively. A cross‐kingdom SynCom comprising the AMF *Rhizophagus irregularis* and the bacterium *Devosia* sp. ZB163 enhanced N and P uptake and increased plant biomass in *Prunella vulgaris* through synergistic interactions [151]. Concurrently, SynComs can enhance crop stress tolerance by modulating plant transcriptomic responses [127]. For example, a SynCom comprising *Pseudoduganella* sp. 1H4, *Pseudoduganella* sp. 9C5, *Asticcacaulis* sp. 9G4, *Ensifer* sp. 10D7, *Massilia* sp. 22G3, *Ensifer* sp. 10C12, and *Caulobacter* sp. 25H3 suppressed the expression of osmotically induced oxidative stress genes and activated aquaporin genes, enhancing root water uptake capacity in *Arabidopsis* [152]. Furthermore, SynComs can function by restructuring the rhizosphere microbial community [153]. For example, in pepper cultivation, a SynCom composed of *Bacillus subtilis*, *Trichoderma harzianum*, *Trichoderma asperellum*, and *Aspergillus* sp. modulated the rhizosphere microbial community structure and increased the abundance of key taxa, improving nutrient acquisition, stress resilience, and pathogen resistance [105]. This promoted pepper growth, significantly increasing stem height (20.9%), stem diameter (36.33%), fresh weight (68.84%), dry weight (64.34%), chlorophyll content (29.65%), leaf number (27.78%), root activity (117.42%), root tip number (35.4%), total root length (21.52%), and root specific surface area (39.76%).

Microorganisms can inhibit pathogens through direct antagonism, such as niche competition and antimicrobial compounds secretion [154, 155]. As mentioned above, SynComs can enhance plant defense by ISR; for example, *Trichoderma harzianum* OMG16 and *Bacillus velezensis* FZB42 synergistically activate defense responses against *Verticillium longisporum* in oilseed rape, enhancing disease resistance by 100‐fold [156]. A SynCom has also been shown to effectively treat *Astragalus* root rot by activating ISR, reducing disease incidence by 42.7% [104]. Furthermore, a cross‐kingdom SynCom composed of 10 strains of bacteria and fungi exhibited remarkable efficacy in suppressing tomato fusarium wilt, reducing incidence by 60%, potentially linked to the modulation of plant immunity and microbial interactions [157]. SynComs also offer novel approaches for integrated root disease management. Carrión et al. (2019) discovered that pathogen‐induced endophytic microbiomes activate disease‐suppressive functions, enriching specific bacterial taxa and genes associated with suppression [158]. These successful case studies demonstrate that SynComs inhibit plant diseases and enhance soil health through mechanisms including antimicrobial metabolite production, surfactin secretion, synergistic growth promotion, and immune activation.

### 4.3 Environmental Pollution Remediation

SynComs also demonstrate significant potential in the field of contaminant biodegradation, with different consortia exhibiting distinct functionalities and degradation mechanisms. Research on SynCom‐based bioremediation of traditional pollutants, including herbicides, heavy metals, and petroleum hydrocarbons has been extensive [159, 160, 161, 162, 163, 164]. For example, a SynCom comprising *Pseudomonas* sp. ADP and *Arthrobacter* sp., achieving 95% degradation of atrazine with the initial concentration of 100 mg/L within 7‐days under lab conditions [159]. Additionally, a SynCom consisting of *Enterobacter* sp. A11 and *Comamonas* sp. A23, which reduced the average cadmium (Cd) content in plant shoots by 71.3% [160]. Furthermore, bacterial‐fungal SynComs, such as a combination of the white‐rot fungus *Phlebia brevispora* with plant growth‐promoting bacterial strains *Enterobacter* sp. TN3W‐14 and *Pseudomonas* sp. TN3W‐8, significantly enhanced the PAH‐degradation capacity of *P. brevispora*, achieving 86% degradation of pyrene and 53% degradation of benzo[a]pyrene within 15 days [161]. Additionally, a SynCom of *Acinetobacter baumannii* and the fungus *Talaromyces* sp. achieved 80% PAH degradation within 14 days [162]. Within this system, the fungus exhibited superior capability for degrading n‐alkanes, while the bacterium more effectively degraded aromatic hydrocarbons and branched alkanes. A combination of the fungus *Fomitopsis pinicola* and the bacterium *Ralstonia pickettii*, achieved 61% degradation of dichlorodiphenyltrichloroethane (DDT) [163]. Here, *F. pinicola* converted DDT into products utilizable by *R. pickettii*, while *R. pickettii* stimulated the hyphal growth of *F. pinicola. In addition, the significant advantage and* the greatest potential of SynComs in bioremediation lies in the rapid co‐degradation of complex pollutants, reducing the metabolic burden on single strain and accomplishing difficult tasks. For example, a SynCom composed of *Pseudomonas* sp. S1, *Psychrobacter* sp. S2, *Arthrobacter* sp. S3, and *Bacillus* sp. S4 could efficiently degrade eight herbicides, achieve stable colonization, increase soil bacterial biodiversity, and alter soil enzyme activity. Meanwhile, strains S1, S2, and S4 mainly perform C metabolism and energy metabolism functions, such as the tricarboxylic acid cycle, pyruvate metabolism, glycolysis, and glycine metabolism, and strain S3 mainly performs herbicides degradation, demonstrating effective metabolic division of labor among members [15]. Furthermore, the consortium of *Pseudomonas putida* C2 and *Paenarthrobacter ureafaciens* AT achieved co‐degradation of disparate pollutants, removing 95% of hexavalent chromium (Cr(VI)) and 100% of atrazine within 5 days [164].

Emerging contaminants [165] include antibiotics (e.g., sulfonamides), microplastics (e.g., polyethylene (PE), polypropylene (PP)), endocrine disruptors (e.g., bisphenol A (BPA), phthalate esters (plasticizers)), perfluorinated substances (PFAS, e.g., perfluorooctane sulfonate (PFOS), perfluorooctanoic acid (PFOA)), and antibiotic resistance genes (ARGs) [166]. Bioremediation of these emerging contaminants faces significant challenges, including weak degradative capacity, difficulties in breaking strong C‐F bonds, incomplete degradation leading to secondary pollution, and enhanced mobility [167]. Consequently, single microbial strains are often inadequate for efficient and stable remediation. A SynCom comprising *Rhodococcus jostii*, *Pseudomonas putida*, and two metabolically engineered *Bacillus subtilis* strains degraded 31.2% of a polyethylene terephthalate (PET) film within 3 days under lab conditions [168]. Another SynCom, consisting of *Glutamicibacter* sp. ZJUTW, *Cupriavidus* sp. LH1, and *Gordonia* sp. GZ‐YC7, was capable of simultaneously and efficiently degrading six priority phthalate esters in minimal salts medium and in soil [169]. The SynCom completely degraded dimethyl phthalate (DMP), diethyl phthalate (DEP), benzyl butyl phthalate (BBP), and dibutyl phthalate (DBP) within 6 days in soil, and 70.84% of di(2‐ethylhexyl) phthalate (DEHP) and 66.24% of dinoctyl phthalate (DOP) within 2 weeks. In summary, research on the biodegradation of emerging contaminants using SynComs is promising but remains relatively limited.

### 4.4 Carbon Sequestration and Emissions Reduction

C sequestration and emissions reduction, specifically enhancing soil organic carbon (SOC) and minimizing SOC loss, play a vital role in improving soil physical structure, enhancing water and nutrient retention capacity [170]. While soils harbor diverse microorganisms capable of C fixation, the sequestration capacity of individual strains is often limited [171]. SynComs are emerging as promising tools for enhancing soil C sequestration. SynComs based on photoautotrophs can pair cyanobacteria or microalgae as primary C fixation engines with heterotrophic bacteria [172]. The C‐fixing organisms fix CO_2_ into organic matter, such as sugars and acids, which is provided to heterotrophic bacteria. In turn, the heterotrophs, upon consuming this organic matter, supply microbial or small‐molecule growth factors that support the rapid growth and stable function of the C fixers [173]. For example, a synthetic modular co‐culture system utilizing the microalga *Chlamydomonas reinhardtii* and *Escherichia coli* was developed, where *C. reinhardtii* converts CO_2_ to glycolate, and *E. coli* then metabolizes it into valuable bioproducts. Two‐stage continuous co‐culture improved *E. coli* growth and boosted the microalga's C fixation capacity by 30% [172]. Concurrently, leveraging the C fixation abilities of chemoautotrophs (e.g., hydrogen‐oxidizing or sulfur‐oxidizing bacteria) or electroactive microbes (e.g., *Geobacter sp. and Shewanella sp*.), SynComs can be constructed with heterotrophs or methanogens to perform biological C fixation using hydrogen, reduced sulfur compounds, or electrons supplied from electrodes [174]. For instance, a SynCom achieved >90% C fixation efficiency by converting CO_2_ to acetate via microbial electrosynthesis under moderate salinity conditions. Furthermore, strains expressing highly efficient carbonic anhydrase can accelerate CO_2_ hydration to HCO_3−_, and when combined with urease‐producing strains or other carbonate‐precipitating microbes, enable the conversion and sequestration of CO_2_ into stable inorganic carbonates (e.g., CaCO_3_) [175, 176]. Optimized consortia mineralization systems can achieve C fixation rates several orders of magnitude faster than natural processes [177]. It is noteworthy that biological C fixation regulated by synthetic microbial communities occurs not only in soil but can also play a significant role in aquatic environments and other ecosystems. For example, one study established a microalgal‐bacterial SynCom in a wastewater treatment system that not only efficiently removed pollutants like COD, N, and P but also achieved net C fixation through microalgal photosynthesis [178]. Collectively, this evidence demonstrates that SynComs can significantly enhance C fixation efficiency through diverse interaction mechanisms, offering a highly promising biotechnological pathway for C sequestration and emissions reduction.

SynComs can mitigate greenhouse gas emissions and protect the stability of soil ecological functions through mechanisms like cross‐feeding and competitive substrate consumption. For example, a SynCom containing efficient methanotrophs *Methylomonas sp*. and acetogenic bacteria *Clostridium sp*. was constructed. The former directly oxidizes CH_4_, while the latter competitively consumes hydrogen through acetogenesis, which as a substrate for methanogens. Field simulation experiments with application of a SynCom comprised of these organisms showed a 40–60% reduction in CH_4_ emissions without compromising rice yield [179, 180]. Cooperation between AMF and N_2_O‐reducing *Pseudomonas* bacteria living on mycelium significantly reduced N_2_O emissions. Carboxylates exuded by mycelium act as attractants in recruiting *Pseudomonas Fluorescens*, while also serving as stimulants for *nosZ* gene expression [181].

## 5 Challenges and Prospects

Although SynCom research has progressed in areas such as agriculture and environment, the technology still faces numerous challenges [3, 6, 8, 12, 18]. These include environmental adaptability, functional stability, limitations of key technologies, and potential ecological risks. Future research should prioritize the following aspects to advance the widespread application of SynComs in soil health and ecological security.

### 5.1 Environmental Adaptability and Functional Stability

SynComs often work well in the lab but fail when deployed into the field. This failure is often linked to a lack of physicochemical adaptation and reduced metabolic stability when deployed in a complex system. This is primarily driven by four limitations. First, a SynCom is a living, growing community of organisms that will naturally interact with endogenous microbes in the substrate of deployment. This leads to disruption of SynCom, as inoculating concentrations shift due to differences in growth rates between species. Faster‐growing taxa will deplete specific nutrients under co‐culture conditions, resulting in nutrient insufficiency and functional impairment of slower‐growing members [18, 40]. Second, some species that do well in the lab fail to survive in the field, due to unpredictable responses to physicochemical properties such as temperature, pH, nutrient availability, and oxygen potential; in tandem with biological pressures such as competition from endogenous microbes, host immunity, and phage predation, it is surprising that many SynCom strains survive at all [182]. Third, unpredicted metabolic interactions between SynCom members can result from microbial responses to complex systems; for example, inactivation of key metabolism under environmental stress, or degradation of quorum‐sensing signal molecules by environmental enzymes can result in SynCom dysfunction [3]. Finally, SynCom members may mutate or adapt through other means resulting in gene or plasmid loss, which can degrade the functional activity of the SynCom [183].

Therefore, research should not only focus on improving culture conditions to increase the taxonomic and functional breadth of strains available for SynCom design; also optimizing of modeling and analysis of in situ responses for deployed SynComs is required. Designing SynComs without assessing how they will respond in a specific ecosystem greatly limits the effectiveness of these technologies. In addition, innovative deployment strategies can be used, such as a C‐based encapsulation of SynCom members, to facilitate inter‐strain communication and metabolite exchange while protecting the strains from the physicochemical and biological factors that would degrade their potential [40]. To combat environmental stress, genetic engineering approaches introducing inducible expression systems and antioxidant enzymes, can enhance consortium resilience [182, 184]. AI and ML can also be deployed to improve the construction of SynComs so as to reduce the potential for metabolic failure [3, 185].

### 5.2 Technological Bottlenecks

Approximately 70% of microbes in any environmental sample have yet to be effectively cultivated and isolated in a lab, presenting a major throughput bottleneck in strain isolation and screening [9]. Meanwhile, imperfect predictive models for community interactions hinder in‐depth analysis of SynCom interaction networks and member functions, which easily lead to community instability and functional failure upon environmental introduction. Studies revealed that GEMs struggle to simulate higher‐order interactions, such as tri‐species competition, with prediction errors exceeding 40% for substrate competition or toxin accumulation [34, 35, 186]. Conventional metagenomics provides only relative abundance data, failing to quantify the absolute abundance of key functional microbes, resulting in inaccurate strain proportion determination during SynCom design. For example, SynComs designed on traditional metagenomics exhibit colonization failure rates exceeding 60% in the rhizosphere [182]. Additionally, the scarcity and lag in genetic tools severely impede the engineering of non‐model microbes, such as the absence of genetic toolkits and low gene editing efficiency [186]. Research suggests that many potential SynCom strains, such as the high cellulose‐producing *Kosakonia oryzendophytica*, lack standardized genetic tools [184]. Regulatory elements, such as promoters and ribosome‐binding sites, exhibit a narrow range of expression strength (1.84%–169%), making precise metabolic flux control difficult. The widely used CRISPR‐Cas9 technology shows editing efficiencies below 30% in anaerobes like *Clostridium* sp., coupled with low vector transformation rates [186]. More importantly, the functional validation of most SynComs is conducted under laboratory conditions [159, 168]. While a few SynCom formulations have been tested in small‐scale natural environments, significant hurdles remain for large‐scale deployment and industrialization [156, 157, 158, 159, 160, 161, 162, 163, 164, 175]. Research suggests that a disconnect exists between upstream design and midstream process development of SynComs. For instance, 60% of high‐yield strains identified in laboratory shake flasks fail in fermenters, primarily because the parameters such as shear stress and oxygen gradients in shake flask poorly simulate real fermentation [187]. The slow development of high‐throughput fermentation platforms impedes SynComs industrialization. Traditional reactors handle fewer than 50 test batches per year, and parallel bioreactor systems, such as the Manson Bio solution, require expensive automated sampling and detection robots, costing over 2 million RMB per unit. Concurrently, the shortcomings of immobilization and amplification processes can also lead to delays in the large‐scale production of SynComs. Current methods often employ C‐based materials or alginate for microbial encapsulation and immobilization to enhance environmental stability [188]. However, non‐living materials used for encapsulation have high mass transfer resistance, and metabolic byproducts produced by the microbial community, such as hexanoic acid, can degrade the encapsulation material, leading to the destruction of the immobilized structure and a decline in efficiency.

In the future, the development and innovation of SynCom technology should be focused on design tools, stability regulation, function expansion, and industrial application. First, AI‐driven design tools for higher‐order interactions prediction and intelligent design should be developed. On the one hand, integrating genomic, metabolomic, spatial transcriptomic, and other multi‐omics data to draw dynamic maps of microbial interactions, building a multi‐omics fusion colony design platform, combining with AI algorithms to predict the optimal partner of the colony, and constructing synthetic colonies with strong complementarity, high stability, and significant functions. For instance, the BacterAI 2.0 platform can autonomously generate experimental protocols through reinforcement learning, optimizing microbial compositions with only one‐tenth of the data required by traditional methods, and iteratively designing efficient SynComs [36]. In addition, platforms like Super Community Combinations (SuperCC) can predict inter‐strain metabolite exchange networks with high accuracy (>92%), and successfully design SynComs capable of stably degrading PAHs and herbicides, with a 40% increase in degradation efficiency [18]. ML algorithms also show significant potential within SynCom and microbial ecology research for predicting complex physicochemical interactions among community members and monitoring compositional dynamics. Integrating ML with computational frameworks like Computational Microbial Ecosystem Technology Simulator (COMETS) holds promise for screening optimally functional SynComs [189]. Specifically, it refers to predicting the spatial self‐organization patterns, population dynamics, and ecosystem functions of SynComs by simulating their dynamic growth, metabolism, and interactions within complex spatiotemporal environments [189, 190]. Meanwhile, systems such as culture automation and machine intelligence (CAMII), in combination with AI and high‐throughput cultivation techniques, have been proven to facilitate taxonomic and morphological characterization during SynCom assembly, providing novel strategies for targeted SynCom screening [162]. On the other hand, AI technologies should be employed to establish multi‐scale models spanning from the cellular to the community level. Microbial community digital twin (MCDT) is a virtual model based on real‐time data that dynamically mirrors the behavior and state of a real microbial community within a digital space [191, 192]. By integrating multi‐omics information, environmental parameters, and computational simulations, it enables closed‐loop “predict‐optimize‐control” management. Examples include integrating GEMs to simulate single‐strain metabolic fluxes, constructing complex *E. coli* models encompassing over 2,000 biochemical reactions, and the whole‐cell model (WCM), which simulates the entire cell division process [34, 35, 193]. Concurrently, in situ sensors, such as Raman spectroscopy and nanoelectrodes, could continuously monitor metabolite concentrations and environmental data, providing precise insights into SynCom growth and metabolic interactions.

### 5.3 Developments and Future Innovations

Building upon this, future research should prioritize the development of dynamic stability enhancement technologies for SynComs, which aim to facilitate the formation of more robust consortia and mitigate the risk of destabilization under environmental stress. For instance, optogenetically controlled CRISPR‐based switches can be employed to repress inter‐strain competitive genes, thereby enhancing the stability of SynComs. The crotonyl‐CoA/ethylmalonyl‐CoA/hydroxybutyryl‐CoA (CETCH) cyclical microbial community can split the artificial C fixation pathway into three microorganisms, achieving a C dioxide fixation rate of 1.2 g/L/h, which is threefold higher than natural pathway [194, 195]. To ensure the successful deployment of SynComs within complex and fluctuating ecological niches, establishing host‐SynCom simulation systems is a critical future priority, which would enable the adaptive fine‐tuning of SynComs structure and function in response to specific environmental pressures. This capability is essential for realizing a targeted SynComs application aimed at improving soil health, ecological function, and human health. Furthermore, advancements in computational modeling now enable the near real‐time prediction of soil microbial dynamics [189, 193]. Future applications leveraging these predictive models will significantly augment our capacity to address ecological challenges within complex ecosystems. Finally, the advancement of scalable and industrializable technologies for SynComs is a decisive factor in determining whether such communities can function effectively in real‐world applications. Future scale‐up and industrialization of biotechnology must be founded upon principles of sustainability, high efficiency, and low cost. Consequently, developing novel cultivation techniques to enhance SynCom biomass while reducing energy consumption is paramount. Microfluidic technology has emerged as a promising approach within SynCom research, owing to its operational simplicity, high throughput, and inherent stability. Its core principle involves constructing nanoscale cultivation chambers on integrated chips to achieve rapid single‐cell sorting. In the future, microfluidic technology can be further developed and innovated in areas such as precise microbial community construction, high‐throughput screening, single‐cell manipulation, and chip‐based simulation of dynamic environments, which can achieve ultra‐stable microdroplets, enhancing the precision and controllability of droplet throughput, enabling innovative customization of microfluidic devices, and standardizing droplet handling operations. Additionally, more phenotypic testing sensors suitable for droplet systems of different scales need to be developed to significantly enhance phenotypic characterization capabilities, construct multi‐dimensional genotype‐phenotype models, and provide more powerful enabling tools for the application of droplet microfluidic technology in the DBTL cycle [196, 197, 198]. Finally, bio‐sensors such as microelectrode arrays can be established for SynComs during production and functional performance to enable real‐time monitoring and early warning of microbial growth, fermentation, and functional performance, ensuring the stability of microbial production and functionality.

### 5.4 Ecological Risks

While SynComs demonstrate significant promise for application, their potential ecological risks cannot be ignored. First, the potential for a SynCom to invade, dominate and disrupt an ecosystem cannot be ignored [2, 3, 4]. SynComs released into the environment may exhibit unintended persistence or proliferation, potentially displacing native microbial communities by competing for resources and niche space. This could, in some cases, lead to soil nutrient depletion and suppression of beneficial endogenous microorganisms. Next, as foundational components of ecosystems, alterations in microbial composition and function can trigger cascading effects through the food web, progressively impacting plant and animal communities and ultimately destabilizing ecosystem equilibrium [109, 110]. In addition, SynComs may perform unintended functions or metabolize harmful substances within complex ecological environments, resulting in functional dysregulation [18]. At the genetic level, engineered genetic elements such as antibiotic resistance genes within SynComs could disseminate via plasmids, viruses, or other vectors through horizontal gene transfer [130]. This poses risks of genetic pollution, ecosystem disruption, and the potential breakdown of ecological barriers. More importantly, there are inherent difficulties in achieving physical isolation between SynCom application sites, such as farmland, and natural ecosystems [52, 53]. Functional SynComs may migrate via groundwater into adjacent aquatic systems, resulting in cross‐system transmission and the risk of unintended species dispersal and contamination. Consequently, alongside technological innovation, the parallel development of predictive ecology is imperative. We need to be able to predict how an ecosystem responds to an intervention. Therefore, developing digital twins of affected environments will be vital if we are to balance the potential of SynCom technology with ecological safety.

### 5.5 Human Health

Human health represents a critical expression of ecosystem function, reflecting the balance between host physiology and its associated microbial consortia [199]. Within this ecological continuum, SynComs provide a powerful means to reconstruct and stabilize disrupted microbiomes, thereby restoring essential symbiotic functions that sustain metabolic, immune, and neuroendocrine homeostasis. Early applications of SynComs have focused on the gut, where defined consortia of commensal bacteria can correct dysbiosis associated with chronic inflammatory and metabolic diseases. Combinations of *Bifidobacterium longum* subsp. *infantis* and *Faecalibacterium prausnitzii*, for instance, have been shown to reduce intestinal inflammation, strengthen epithelial barrier integrity, and normalize short‐chain fatty acid and bile acid metabolism, mechanisms directly relevant to inflammatory bowel disease and metabolic dysfunction [200, 201, 202, 203]. In neonatal systems, SynComs that mimic the ecological and functional properties of the maternal microbiome are being developed to promote immune tolerance and growth, building on the success of maternal fecal and vaginal microbiota transfer in establishing a stable infant gut microbiome [199].

At other body sites, such as the vaginal and urinary microbiomes, SynCom‐based interventions offer new approaches to prevent or reverse dysbiosis. Engineered consortia dominated by *Lactobacillus crispatus* or *L. jensenii* are being investigated for treating bacterial vaginosis and recurrent urinary tract infections, demonstrating that ecological restoration through microbial community design can provide a sustainable alternative to antibiotic prophylaxis [204, 205]. The potential of SynComs extends beyond site‐specific restoration toward the development of next‐generation ecological probiotics capable of producing targeted immunomodulatory and metabolic functions, ranging from type I interferon regulation and bile acid transformation to neuroactive metabolite synthesis [199]. By integrating synthetic biology, multi‐omics, and computational modeling, future SynComs can be rationally designed to maintain host–microbe homeostasis, offering personalized yet ecologically grounded solutions to human disease. In this sense, SynComs bridge microbial ecology and medicine, representing both a tool for understanding the rules of microbial coexistence and a translational framework for restoring ecosystem health within the human body.

## 6 Summary

SynCom technology has progressed from conceptual demonstrations to an emerging framework for ecological engineering, offering transformative solutions for soil regeneration, sustainable agriculture, environmental restoration, and human health. Advances in modular community design—particularly those leveraging cross‐species metabolic division of labor, adaptive environmental sensing, and multi‐kingdom interactions—are shifting bioengineering from single‐strain optimization toward programmable microbial ecosystems. The next frontier lies in coupling these experimental systems with computational modeling and AI‐driven network inference to decode the vast functional potential of uncultured microbial taxa and expand the design space for synthetic ecosystems. Equally critical is the establishment of closed‐loop pipelines that integrate genetic design, in situ validation, and ecological feedback to ensure functionality, stability, and safety across environments. Through these convergent efforts, SynComs can evolve from experimental tools into scalable technologies that enhance ecosystem resilience and deliver sustainable benefits for both planetary and human health.

## Conflicts of Interest

The authors declare no conflicts of interest.

## Data Availability

The authors have nothing to report.
